# Long-Term Nucleos(t)ide Analogues Therapy for Adults With Chronic Hepatitis B reduces the Risk of Long-Term Complications: a meta-analysis

**DOI:** 10.1186/1743-422X-8-72

**Published:** 2011-02-15

**Authors:** Qin-Qin Zhang, Xuan An, Ying-Hong Liu, Shi-Ying Li, Qing Zhong, Jing Wang, Huai-Dong Hu, Da-Zhi Zhang, Hong Ren, Peng Hu

**Affiliations:** 1 Department of Infectious Diseases, Institute for Viral Hepatitis, Key Laboratory of Molecular Biology for Infectious Diseases, Ministry of Education, The Second Affiliated Hospital of Chongqing Medical University, Chongqing, PR China

## Abstract

**Background:**

The effect of antiviral therapy in chronic hepatitis B (CHB) on reducing the risk of long-term complications (LTCs) remains unclear so far. To study whether long-term nucleos(t)ide analogues therapy can reduce the risk of long-term complications.

**Methods:**

We searched MEDLINE, EMBASE, OVID, the Cochrane Central Register of Controlled Trials. Relative risks (RRs) of long-term complications with or without treatment were studied. Also subgroup analyses including the status of drug-resistance, HBeAg and pre-existing compensated cirrhosis were done using relative risks of long-term complications either with or without treatment or among nucleos(t)ide analogues treatment groups.

**Results:**

Six eligible studies (3644 patients in all) were included. Data showed the incidence of long-term complications in treatment groups was induced by 74%(RR:0.26, 95% CI: 0.15-0.47) compared with no treatment. Whether drug-resistant happened or not during the long-term therapy, the incidence of long-term complications was still significantly induced respectively by 45%(RR: 0.55,95%CI:0.40-0.76) and 78% (RR:0.22, 95%CI: 0.13-0.36). For both different status of HBeAg and pre-existing compensated cirrhosis, there was significant lower incidence of long-term complications in treatment groups compared with no treatment, too. Moreover, among the NA treatment groups, patients with drug-resistance had 2.64 times (RR:2.64, 95%CI: 1.58-4.41) higher chance of developing to long-term complications, and patients with pre-existing compensated cirrhosis also had 3.07 times (RR:3.07, 95%CI: 1.04-9.11) higher chance of developing to long-term complications.

**Conclusions:**

Long-term nucleos(t)ide analogue therapy for adults with CHB prevents or delays the development of long-term complications including decompensated cirrhosis, CHB-related death or CHB-related HCC in patients with CHB. The patients who need take antiviral drugs should receive the antiviral therapy as soon as possible.

## Background

HBV infection is a common global public health problem which affects over 400 million people worldwide [1]. It not only leads to a wide spectrum of liver disease ranging from acute hepatitis (including fulminant hepatic failure) to chronic hepatitis [2] but also the main reason of fatal complications including decompensated cirrhosis and CHB-related HCC that cause up to one million HBV carriers dying of HBV associated liver disease annually [3]. According to natural history, around 15-20% of CHB patients develop cirrhosis in 5 years of follow-up [4,5]. Those with chronic active hepatitis and cirrhosis on liver biopsy have a 5-year survival rate of only 55% [6]. The final goal of treatment for CHB is to induce decompensated cirrhosis, CHB-related HCC or CHB-related death. Two studies more than 3500 CHB patients in Taiwan show the risk of developments of HCC and cirrhosis increases when HBV rapidly replicates, especially after adjustment for sex, age, alcohol consumption, smoking, HBeAg status, serum ALT level and liver cirrhosis [7,8]. It means that suppress of HBV replication may reduce the risk of long-term complications of CHB infection and improve prognosis. Based on this, all guidelines share a common principle regarding nucleos(t)ide analogues treatment for CHB: long-term viral suppression by the drugs with potent antiviral activity and low rate of drug resistance to achieve ''durable response'' to prevent hepatic decompensation, reduce or prevent progression to cirrhosis and/or HCC, and prolong survival [9-11].

There are two kinds of oral antiviral agents approved to treat hepatitis B. Nucleoside analogues include lamivudine, telbivudine and entecavir, while nucleotide analogues include adefovir and tenofovir. They all suppress the replication of HBV in the liver. Studies have shown that treatment of CHB with nucleos(t)ide analog would not only suppress the viral replication but also reduce fibrosis in the liver [12-18]. These findings have changed the concept that fibrosis is irreversible. Consensus has been reached that treatment must be often administered long-term as the high rate of virological relapse when nucleos(t)ide analogue therapy is discontinued. However, long-term therapy may increase the emergence of resistant viral variants and sometimes may be associated with hepatitis flares, which may compromise the initial clinical benefit of the treatment. So far the effect of treatment on reducing the risk of long-term complications (LTCs) remains unclear. On the other hand, whether drug-resistant mutation is associated with high risk of developing long-term complications remains another open question. The major reasons include relatively small sample size, lack of adequate controls, short time of follow-up, different age groups at enrolment and so on.

Traditionally, meta-analysis is applied and best confined to RCTs. However, there were NRCTs [19,20] chosen into a meta-analysis before when controlled placebo groups were difficult to perform in the clinical.

According to the available evidence and assessing it by meta-analysis, we made an effort to compare the effect of NA therapy vs. no therapy in the incidence of long-term complications of CHB on the basis of published data. We also attempted to study the effects of HBeAg status, pre-existing compensated cirrhosis, virological response to NA and drug-resistance to NA on the risk of long-term complications.

## Methods

We searched MEDLINE, EMBASE, OVID, the Cochrane Central Register of Controlled Trials [21] using keywords "(nucleoside analog OR lamivudine OR entecavir OR adefovir OR telbivudine OR tenofovir) AND (hepatitis B OR HBV) AND (cirrhosis OR carcinoma OR cancer OR hepatic failure OR die OR death)". The limits were "human" and "English". We included all randomized controlled trials, case-control or cohort studies of adults with CHB published in English between Jan.1998 and Jul.2010, if they met the following criteria:

(i) Confirmed CHB infection including CHB and early cirrhosis whose definition was described clearly in the articles.

(ii) Either NA (lamivudine, adefovir, entecavir, telbuvidine, tenofovir) was used for treatment.

(iii) In randomized-control trial (RCT) a control group with placebo or no antiviral treatment, and in case-control or cohort study the inclusion of a control group with no antiviral treatment.

(iv) The final outcome of studies was the number of incidence of long-term complications such as CHB-related death, CHB-related HCC or decompensated cirrhosis diagnosed during the period of study in both treatment and control groups, and the following-up time was more than 2 years.

Patients were excluded if:

(i) Studies with duplicated publications on the same group of patients, including those reported early stages of study findings within complete sample size.

(ii) They were children, or pregnant women, or had several causes of hepatitis such as HCV/HDV/HIV/autoimmune hepatitis and so on, unless outcomes for participants meeting our eligibility criteria were reported separately.

(iii) They had decompensated liver disease or hepatocellular carcinoma at baseline.

(iv) They had received chemotherapy any systemic antiviral therapy, immunomodulators, cytotoxic agents, or corticosteroids within 6 months or liver transplantation before.

(v) Studies with less than 50 cases.

We tried to analysis the various factors which effect the main outcomes including pre-existing compensated cirrhosis, drug-resistance to anti-viral agents, HBeAg status and virological response to treatment on long-term complications development. Studies lacking clear definitions of cirrhosis, drug resistance and virological response were not included in subgroup analysis. Pre-existing compensated cirrhosis was referred as Ischaks score of 5-6 and radiological and/or histological diagnosis of cirrhosis without evidence of decompensation of hepatic function and portal hyperion. Drug-resistance was defined as reappearance of HBV DNA after initial negativity. Virological response was defined as disappearance of HBeAg with or without appearance of anti-HBe and undetectable HBV DNA levels.

### Denition of long-term complications

Long-term complications of CHB are the end events of clinic including decompensated cirrhosis which included occurrence of ascites, esophageal/gastric variceal bleeding, spontaneous bacterial peritonitis or hepatic encephalopathy; HBV-related HCC diagnosed histologically or by diagnostic imaging method(s), given an alpha fetoprotein (AFP) level above 400 ng/mL; or HBV-related death.

### Quality assessment

Quality of each study was assessed based on following criteria:

(i) For RCT: random sequence generation, allocation concealment, blind method, and description of withdrawals and dropouts. The qualities of the first three items were classified into three grades respectively: adequate (2 points), unclear (1 point), and inadequate (0 point). And the fourth item was classified into two grades: described (1 point) and not described (0 point). The scores of modified Jadad quality scale were ranged from 0 to 7, with scores ≥ 4 signifying high-quality studies.

(ii) For case-control study: case-matched by patient's characteristics (age, gender, hepatitis function, percentage of pre-existing cirrhosis and HBeAg-positive, and so on).

(iii) Definitely listing of inclusion and exclusion criteria for patients.

(iv) Clear definitions of treatment response (drug-resistance and virological response) and long-term complications diagnosis.

### Statistical analysis

Meta-analysis was performed using fixed-effect or random-effect methods, depending on absence or presence of significant heterogeneity [22]. Chisquared test was used to test whether statistical heterogeneity existing or not and we considered it to have existed when P < 0.10. Statistical heterogeneity was also assessed with I^2^, which indicates the percentage of total variation across studies. The random-effect model would be used if there was significant heterogeneity, otherwise the fixed-effect model would be used. We used the relative risk(RR) of the main outcomes as the measure of efficacy. The 95% confidence interval(CI) for the combined RR is also provided. Analyses were performed with review manager version4.2.2 (RevMan, The Cochrane Collaboration, Oxford, England)

## Results

### Characteristic and quality of studies

**Figure 1:**
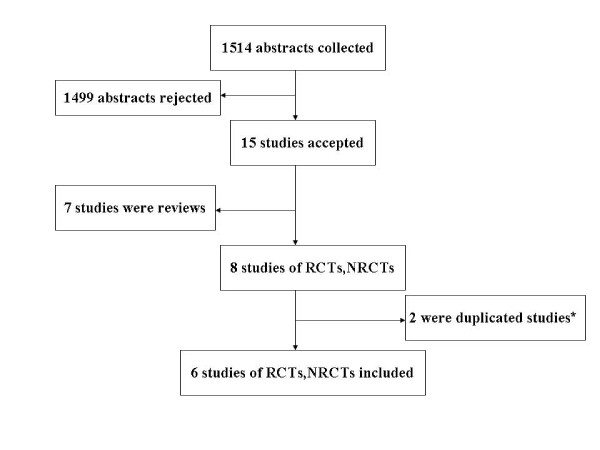
**Results of literature search**.* Three studies were published from the same group of investigators [23,29,30]. Only the most updated publication with a completed dataset was included.

1514 abstracts were found, among them eight studies [23-30], of RCTs and NRCTs eligible in this meta-analysis excluding seven reviews. There were three[23,29,30]repeated publications at different time points and we selected only one. The final analysis therefore included six studies published from 2004 to 2010[23-28] (Figure 1). The main features of the studies evaluated by meta-analysis are shown in Table 1. All studies on antiviral agent used lamivudine[23-28]and two study added (or switched to) adefovir in case of lamivudine resistance[24,27].

**Table 1:** Characteristics of included clinical trials in meta-analysis

Study	Region	Study type	NA regimens (/day)	Sample size (n) (treatment vs. placebo)	Mean age (ys) (treatment vs. placebo)	Male(%) (treatment vs. placebo)	Mean ALT(U/L) (treatment vs. placebo)	Pre-existing cirrhosis(%) (treatment vs. placebo)	HBeAg (+) (%) (treatment vs. placebo)	Mean treatment duration (mon)	Follow up period (ys)
Papatheodoridis et al.[24]	Greece	Cohort	LAM 100 or 150 mg then add or switch to ADV	396 (201 vs. 195)	55.9 (52 ± 11) vs. (49 ± 14)	82.6 (83.1 vs. 82.1)	83.2 (98 vs. 68)	33.3 (31.8 vs. 34.9)	0	48	3.8
Matsumoto et al.[25]	Japan	Case-control	LAM 100 mg	754 (377 vs. 377)	41.5 (41.5 ± 12.0) vs. (41.4 ± 12.2)	72.8 (73.2 vs. 72.4)	171.6 (191.7 ± 234.8) vs. (151.5 ± 180.5)	17.5 (17.2 vs. 17.8)	54.8 (51.2 vs. 58.4)	18.9	2.7
Yuen et al.[26]	Hong Kong	Cohort	LAM 100 mg	266 (142 vs. 124)	33.7 (33.9 vs. 33.4)	73.7 (74.7 vs. 72.6)	61 (65 vs. 56.5)	0	100 (100 vs. 100)	89.9	8.2
Liaw et al.[23]	Taiwan	RCT	LAM 100 mg	651 (436 vs. 215)	43.3 (43 vs. 44)	85 (85 vs. 85)	69.3 (70 vs. 68)	61.3 (60 vs. 65)	58 (58 vs. 58)	32.4	2.7
Eun et al.[27]	Korea	RCT	LAM 100 mg then add or switch to ADV	222 (111 vs. 111)	M	M	M	100	M	M	4.4
Jong Ryul Eun et al.[28]	Korea	Cohort	LAM	1355 (768 vs. 587)	36.8 (39.2 vs. 33.6)	M	152.7 (161.3 vs. 141.4)	50.9 (67.3 vs. 25.2)	85.2 (80.1 vs. 91.8)	More than 48	4.4

M: Data missing in the studies; LAM: Lamivudine; ADV: Adefovir dipivoxil

There were 3644 patients in all, 2035 of whom received treatment and 1609 didn't. Among the six studies[23-28], there were two randomized controlled trials[23,27], one case-control study[25]and there cohort studies[24,26,28]. (Table1). One trial was published as an abstract[27]and five published as full publication.

Patients' selection criteria were different among studies. One studies[26]included only patients with HBeAg positive/negative CHB, four studies[23-25,28]included patients with HBeAg positive/negative CHB or Child-Pugh A cirrhosis, while one studies[27] included only patients with HBV-related cirrhosis. The sample size of each study varied greatly, ranging from 222[27]to 1355[28] patients. The mean age ranged from 33.7[26]to 52[24]years old for treated patients and from 33.4[26]to 49[24]years old for untreated patients. The percentage of males ranged from 72.8%[25] to 85%[23]. The length of follow up differed among studies, ranging from 2.7 years[23,25] to 8.2 years[26]. Generally, age, male/female ratio, percentage of pre-existing cirrhosis, and length of follow up were not signicantly different between treated and untreated groups in these studies. But the ALT levels were significantly higher in treated than in untreated groups in four studies[24-26,28].

### Effect of nucleotide/side analogues on development of LTCs

**Figure 2:**
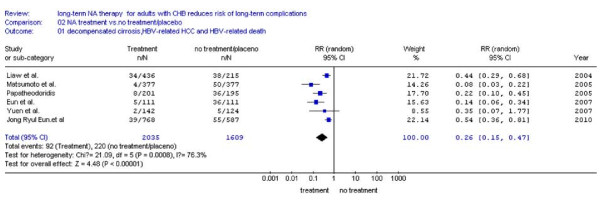
**Forest plot to compare nucleos(t)ide analogues with placebo or no treatment on the incidence of LTCs including decompensated cirrhosis, CHB-related death or CHB-related HCC**.

Data collected from six studies is shown in Table 1. Generally, LTCs occurred in 4.52%(92/2035) in NA treatment patients and 13.7%(220/1609) untreatment patients during following up more than two years. Random effect model was used to combine the results since statistical heterogeneity was found (*I*^*2 *^= 76.3%, *P *= 0.0008 < 0.10) (Figure 2). The combined RR was 0.26,(95% CI: 0.15-0.47), and statistical significant (*P *< 0.00001). These results suggest NA treatment significantly decreased the incidence of LTCs compared with no treatment in a long-term follow-up period.

### Subgroup analysis

To investigate the various factors of effecting the development of LTCs, we applied subgroup analyses including the aspects of drug-resistance, HBeAg status or pre-existing compensated cirrhosis.

### Incidence of LTCs according to the status of drug-resistance

#### Comparation between NA treatment and no treatment

**Figure 3:**
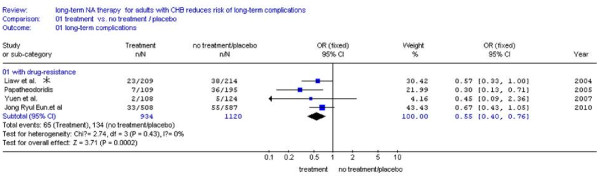
**Forest plots of nucleos(t)ide analogue studies: subgroup analysis on the incidence of LTCs in patients with drug-resistance between NA treatment and no treatment**. Liaw et al * (Figure 3, Figure 4 and Figure 5):two patients had evidence of YMDD mutations at baseline and five patients no samples after baseline, so data on the emergence of YMDD mutations during therapy were available for 644 patients[23].

**Figure 4:**
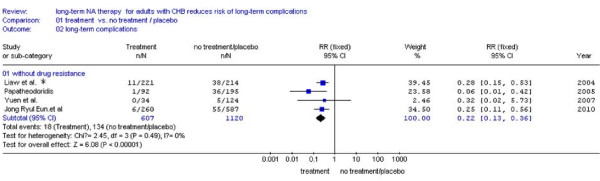
**Forest plots of nucleos(t)ide analogue studies: subgroup analyses on the incidence of LTCs in patients without drug-resistance between NA treatment and no treatment**.

Four studies [23,24,26,28] were included. The remaining two studies[25,27] didn't report the number of patients with or without drug-resistance, and we excluded them. Subgroup analysis with drug-resistance showed a significantly lower incidence of LTCs compared NA treatment (7.0%,65/934) with no treatment (12.0%,134/1120) (RR: 0.55,95%CI:0.40-0.76; Figure 3). Also we found much fewer events of main outcome between NA treatment (3.0%, 18/607) and no treatment (12.0%,134/1121) in the same studies of patients without drug-resistance [23,24,26,28] (RR:0.22, 95%CI: 0.13-0.36; Figure 4). No statistical heterogeneity was found in both Subgroups and fixed effect model was used (Figure 3, *P *= 0.43 > 0.10 and Figure 4, *P *= 0.49 > 0.10).

### Comparation between patients with and without drug-resistant among the NA treatment groups

**Figure 5:**
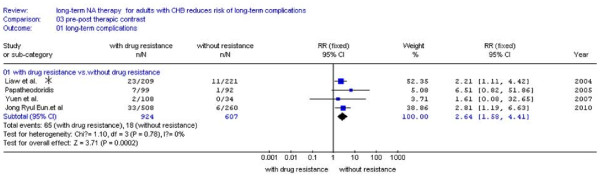
**Forest plots of nucleos(t)ide analogue studies: compared the incidence of LTCs between patients with and without drug-resistant among the NA treatment groups**.

We also tried to find out that whether the factors of drug-resistance could effect the development of LTCs or not among the treatment groups. There were four studies[23,24,26,28] reported the number of patients who occurred LTCs with or without drug-resistance separately. The patients with drug-resistance had high probability of LTCs (7.0%, 65/924) than the patients without drug-resistance (3.0%, 18/607) (Figure 5). No statistical heterogeneity was found and fixed effect model was used (*P *= 0.78 > 0.10, RR:2.64, 95%CI: 1.58-4.41; Figure 5).

### Incidence of LTCs according to HBeAg status

#### Comparation between NA treatment and no treatment

**Figure 6:**
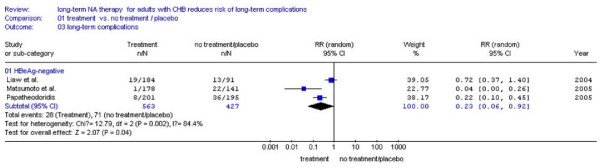
**Forest plots of nucleos(t)ide analogue studies: subgroup analyses on the incidence of LTCs in HBeAg-negative patients between NA treatment and no treatment**.

**Figure 7:**
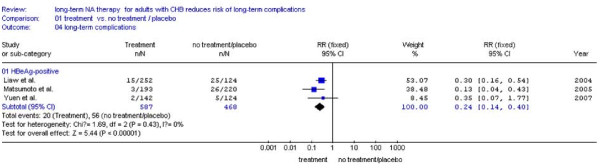
**Forest plots of nucleos(t)ide analogue studies: subgroup analyses on the incidence of LTCs in HBeAg-positive patients between NA treatment and no treatment**.

Three studies[23-25] were included. The remaining one studies[27]was excluded because of lacking the number of HBeAg-positive patients. It was shown that HBeAg-negative patients received NA treatment had hugely lower incidence of LTCs (5.0%,28/563) compared with placebo (16.6%,71/427) (RR:0.23,95%CI:0.06-0.92; Figure 6). Among the HBeAg-positive patients there was a numerical trend of reduced LTCs between the NA treatment (3.4%, 20/587) and no treatment (12.0%,56/468) (RR:0.24, 95%CI: 0.14-0.40; Figure 7). In addition, there was no statistical heterogeneity found and fixed effect model was used (Figure 7, *P *= 0.43 > 0.10).

We also tried to compare the prognosis of patients with different HBeAg status among the treatment groups. Unfortunately, only two studies[23,25] were available and we didn't do the meta-analysis.

### Incidence of LTCs according to the status of pre-existing compensated cirrhosis

**Figure 8:**
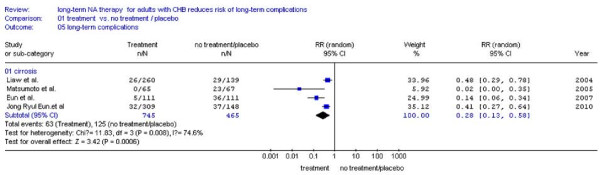
**Forest plots of nucleos(t)ide analogue studies: subgroup analyses on the incidence of LTCs in patients with pre-existing cirrhosis**.

**Figure 9:**
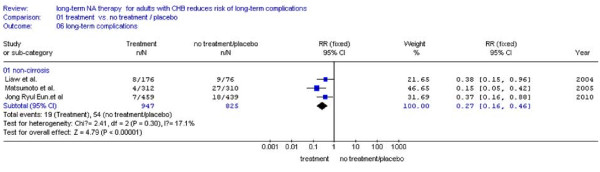
**Forest plots of nucleos(t)ide analogue studies: subgroup analyses on the incidence of LTCs in patients without pre-existing cirrhosis**.

There were four studies[23,25,27,28] reported the number of patients with pre-existing compensated cirrhosis, the remaining one study[24] was excluded because of lacking the number of patients with pre-existing compensated cirrhosis. We found that NA treatment patient had less chance of LTCs (8.5%, 63/745) than the patients without treatment (26.9%, 125/465) (RR:0.28, 95%CI: 0.13-0.58; Figure 8). For patients without pre-existing compensated cirrhosis, incidence of LTCs in NA treatment groups is lower (2.0%, 19/947) than no treatment groups (6.5%, 54/825), too (RR:0.27, 95%CI: 0.16-0.46; Figure 9). No statistical heterogeneity was found, so fixed effect model was used (Figure 9, *P *= 0.30 > 0.10).

### Comparation between patients with and without pre-existing compensated cirrhosis among the NA treatment groups

**Figure 10:**
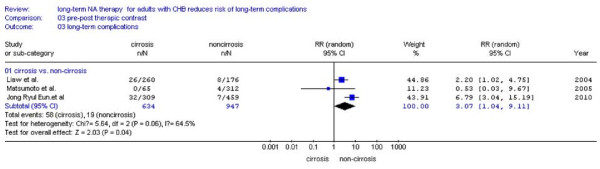
**Forest plots of nucleos(t)ide analogue studies: compared the incidence of LTCs between patients with and without pre-existing compensated cirrhosis among the NA treatment groups**.

Prognosis of patients with and without pre-existing compensated cirrhosis was analyzed among the treatment groups. There were three studies reported the number of patients who occurred LTCs with pre-existing compensated cirrhosis, and these patients had high probability of LTCs (9.1%, 58/634) than the patients without pre-existing compensated cirrhosis (2.0%, 19/947) (Figure 10). Statistical heterogeneity was found and random effect model was used (*P *= 0.06 < 0.10, RR:3.07, 95%CI: 1.04-9.11; Figure 10).

## Discussion

The researchers of the REVEAL study analyzed the relationships between the development of HCC and high HBV DNA load, according to the natural history of chronic HBV infection, and the findings of this study suggested that effective control of HBV replication can reduce the risk of hepatocellular carcinoma in the long run theoretically[7]. However, it is still unclear whether the risk of HCC and other long-term complications can be decreased by the recommendation of medical treatments including interferon and nucleotide/side analogues. Interferon has been used in the treatment of CHB for decades. Lots of studies have been reported the long-term prognosis of interferon, and Y.-F. Yang et al has generalized a conclusion that IFN prevents or delays the development of liver cirrhosis and HCC in patients with CHB [31]. Lamivudine was the first nucleoside analogues used as long-term maintenance therapy since the late 1990s, and other nucleotide/side analogues including telbivudine, entecavir, adefovir and tenofovir were approved recent years. The clinical efficacy and particularly on the incidence of long-term complications under long-term antiviral therapy are difficult to monitor because cirrhotic complications and HCC take long time to develop, especially in noncirrhotic patients whose incidence of liver-related complications is even lower. As a result, in most clinical trials, biochemical response, virologic response and histologic response [32-34] have been used as surrogate end points to determine if the treatment of patients with chronic HBV infection has been successful. Whether these surrogate end points can reflect long-term clinical outcomes was still an open question [35]. Vincent Wai-Sun Wong at al. has confirmed that normalization of serum ALT, HBV DNA suppression, HBeAg loss, or HBeAg seroconversion at the end of drug trials were associated but unable to predict long-term clinical outcomes completely [36]. Even histologic response can't exactly reflect the long-term clinical outcomes as regression of compensated cirrhosis can be translated into favorable clinical outcome in those treated with long-term nucleotide/side analogues therapy[24].

In this meta-analysis, we choose the clinical end points including decompensated cirrhosis, CHB-related death or CHB-related HCC which directly reflect the prognosis of chronic HBV infection to evaluate the efficacy of the long-term nucleos(t)ide analogues therapy.

The most important finding of this meta-analysis is that more than two years NA therapy for adults with CHB reduces the risk of LTCs including decompensated cirrhosis, CHB-related death or CHB-related HCC by pooling data from six studies[23-28].We also analyzed the various factors of effecting the risk of LTCs in some items such as drug-resistance, HBeAg status or pre-existing compensated cirrhosis, and in any item, NA therapy was shown the benefit than placebo of reducing the incidence of LTCs. So for patients with drug-resistance, NA therapy still had a better outcome than the untreated patients with lower risk of LTCs. Among the treated patients, we also investigated whether drug-resistance effected the risk of LTCs. Then it was found that the incidence of LTCs were lower in the non-nucleoside drug resistance groups. At the same time, we compared the prognosis of patients with and without pre-existing compensated cirrhosis among the treatment groups. Then it was found that the incidence of LTCs were lower in patients without pre-existing compensated cirrhosis. It suggested us that the earlier antiviral therapy is been done, the better the prognosis will be. Based on the two conclusions above, the patients who need take antiviral drugs should receive the antiviral therapy as soon as possible. Moreover, and it would be best that drug-resistance genes were detected before patients administrated the antiviral drugs, then proper antiviral therapy could be chosen to reduce the probability of drug-resistance. On the other hand, management of drug-resistance is absolutely important, too. Patients should regularly be followed-up to monitor the drug-resistance. Fortunately, with the available of newer drugs like adefovir dipivoxil without cross resistance to lamivudine [37], the problem of LAM-resistance will go to remission. Currently, add-on adefovir therapy is the most widely accepted strategy in case of LAM-resistance. Chen et al.[38] confirmed the view that the combination of ADV with LAM was superior in inhibiting HBV replication and preventing drug resistance as compared to ADV alone for LAM-resistant CHB patients by a meta-analysis. Other nucleotide/side analogues were available such as entecavir [39-41], tenofovir [42] recent years.

There are several limits in our meta-analysis. Firstly we collected the data from not only randomized controlled trials but also case-control, cohort, and historical controls studies because of very few RCTs. When patients are diagnosed CHB or HBV-related cirrhosis especially with the abnormal liver function and the evidence of HBV replication, it is unethical to running randomized controlled trials. Maybe this is the leading reason that so few RCTs were conducted. Secondly, the length of following up differed among studies, ranging from 2.7 years[23,25]to 8.2 years[26]. As we know, the longer time of following up, the higher incidence of LTCs would be seen. So in our meta-analysis, the incidence of LTCs differed significantly. Maybe this is one reason of statistical heterogeneity. Thirdly, the data we've selected was lacking of testing whether drug-resistance existed or not before therapy because most of the studies didn't show the data about it.

In conclusion, the result of this meta-analysis indicate that long-term nucleos(t)ide analogue therapy for adults with CHB prevents or delays the development of long-term complications including decompensated cirrhosis, CHB-related death or CHB-related HCC in patients with CHB. The patients who need take antiviral drugs should receive the antiviral therapy as soon as possible. During the monotherapy, when LAM-resistance happened, lamivudine should be combined with other antiviral drugs without cross resistance to lamivudine since it still has more benefit than the untreated patients.

## Abbreviations

HBV: Hepatitis B virus; HCC: Hepatocellular Carcinoma; CHB: Chronic Hepatitis B; ALT: alanine aminotransferase; AST: aspartate aminotransferase; LTCs: long-term complications; RCTs: randomized controlled trials; NRCTs: nonrandomized controlled trials; NA: Nucleos(t)ide Analogues.

## Competing interests

The funding source had no influence on study design, in the collection, analysis, and interpretation of the data, in the writing of the manuscript, or in the decision to submit the manuscript for publication. The contents are solely the responsibility of the authors and do not necessarily represent the views of the funding source.

## Authors' contributions

HP and RH conceived the study, provided fund supporting and revised the manuscript critically for important intellectual content. ZQQ made substantial contributions to its design, acquisition, analysis and interpretation of data. AX, LYH, LSY, ZQ, WJ, HHD and ZDZ participated in the design, acquisition, analysis and interpretation of data. All authors read and approved the final manuscript.
